# Targeted clearance of senescent cells using an antibody-drug conjugate against a specific membrane marker

**DOI:** 10.1038/s41598-021-99852-2

**Published:** 2021-10-13

**Authors:** Marta Poblocka, Akang Leonard Bassey, Victoria M. Smith, Marta Falcicchio, Ana Sousa Manso, Mohammad Althubiti, XiaoBo Sheng, Andrew Kyle, Ruth Barber, Mark Frigerio, Salvador Macip

**Affiliations:** 1 Mechanisms of Cancer and Aging Laboratory, Department of Molecular and Cell Biology, University of Leicester, University Road, Leicester, LE1 7RH UK; 2 Department of Biochemistry, Faculty of Physical Sciences, Cross River University of Technology, Calabar, Nigeria; 3 The Ernest and Helen Scott Haematological Research Institute, University of Leicester, Leicester, UK; 4 Leicester Institute for Structural and Chemical Biology, University of Leicester, Leicester, UK; 5 School of Chemistry, University of Leicester, Leicester, UK; 6 Leicester Drug Discovery and Diagnostics, University of Leicester, Leicester, UK; 7 Department of Biochemistry, Faculty of Medicine, Umm Al-Qura University, Mecca, Saudi Arabia; 8 Abzena, Babraham Research Campus, Babraham, Cambridge, UK; 9 FoodLab, Faculty of Health Sciences, Universitat Oberta de Catalunya, Barcelona, Spain

**Keywords:** Senescence, Target identification

## Abstract

A wide range of diseases have been shown to be influenced by the accumulation of senescent cells, from fibrosis to diabetes, cancer, Alzheimer’s and other age-related pathologies. Consistent with this, clearance of senescent cells can prolong healthspan and lifespan in in vivo models. This provided a rationale for developing a new class of drugs, called senolytics, designed to selectively eliminate senescent cells in human tissues. The senolytics tested so far lack specificity and have significant off-target effects, suggesting that a targeted approach could be more clinically relevant. Here, we propose to use an extracellular epitope of B2M, a recently identified membrane marker of senescence, as a target for the specific delivery of toxic drugs into senescent cells. We show that an antibody–drug conjugate (ADC) against B2M clears senescent cells by releasing duocarmycin into them, while an isotype control ADC was not toxic for these cells. This effect was dependent on p53 expression and therefore more evident in stress-induced senescence. Non-senescent cells were not affected by either antibody, confirming the specificity of the treatment. Our results provide a proof-of-principle assessment of a novel approach for the specific elimination of senescent cells using a second generation targeted senolytic against proteins of their surfaceome, which could have clinical applications in pathological ageing and associated diseases.

## Introduction

Senescence is an irreversible proliferation arrest and a key restriction mechanism to prevent the propagation of damaged cells^1,2^. However, the progressive accumulation of senescent cells with time has been associated with loss of tissue homeostasis, and is known to contribute to the functional impairment of different organs typically seen in ageing^3^. Recently, it has been shown that it also plays an important role in fibrosis^4^ and tumour progression^5^, and that it may be involved in cataracts, obesity, diabetes, Alzheimer’s and Parkinson’s diseases, arthritis, atherosclerosis and many other age-related conditions^6,7^. This supports the hypothesis that senescence is an antagonistically pleiotropic process, with beneficial effects in the early decades of life of the organism (in development^8^, tissue repair^9^ and as a tumour suppressor mechanism^10–13^) but detrimental to fitness and survival at later stages, after the percentage of senescent cells in tissues reaches a critical threshold^14,15^. This is thought to be mediated, at least in part, by the secretion of a series of growth factors, chemokines and cytokines, collectively known as the senescence-associated secretory phenotype (SASP)^16,17^.

Consistent with this view, it has been reported that clearing senescent cells from tissues has a protective effect against cancer^5,18^ and the onset of age-related pathologies^19,20^. Because of this, great interest has been placed in a recently discovered group of drugs that can preferentially kill senescent cells, collectively known as senolytics, which have been shown to increase healthspan and lifespan of mice^21^ with attenuation of age-related dysfunctions like emphysema^22^, hepatic steatosis^23^, lung fibrosis^24^, osteoporosis^25^, osteoarthrisis^26^, cardiac regeneration dysfunctions^27^, cognitive memory impairments^28^ or Alzheimer disease^29^ in different in vivo models. Recently, senolytics, were shown to also decrease the number of senescent cells in humans^30^ and alleviate the symptoms of idiopathic pulmonary fibrosis^31^. Despite these important advances, the translational potential of senolytics is still limited, due to its multi-target nature^32,33^. Although other compounds of potential interest are being investigated^34–37^, some of them of natural origin^34,38–42^, a more specific strategy would be desirable in order to reduce the side effects and increase the efficiency of these drugs.

In this context, targeted senolytics are emerging as a promising alternative. For instance, it has recently been shown that toxic nanoparticles activated by the presence of β-galactosidase can eliminate senescent cells in vitro and in vivo, confirming the feasibility of the approach^43^. We propose that the senescent surfaceome, the specific profile of membrane proteins differentially upregulated in senescent cells, could be used to this end even more effectively. Using mass spectrometry, we identified a number of markers highly expressed in the plasma membranes of senescent cells in response to the activation of one of the two main pathways of induction of the phenotype (p53/p21 or 16)^44,45^. Moreover, we showed that molecularly imprinted nanoparticles (nanoMIPs), nanostructured polymeric particles that can recognise and bind a target molecule thus acting like “plastic antibodies”^46^, can bind to an extracellular epitope and thus detect and kill senescent cells^47^, providing the first evidence that the senescent surfaceome can be used to design targeted senolytics^48^.

It was recently shown that antibody-dependent cell-mediated cytotoxicity can be used to eliminate senescent cells by activating NK cells using the senescent surfaceome^49^. We propose that a similar principle could be used to design antibody–drug conjugates (ADCs) to deliver drugs into senescent cells by binding to an extracellular epitope of the surfaceome. ADCs are monoclonal antibodies attached to biologically active cytotoxic drugs via specific linker molecules^50^. The ADCs recognize and bind to an antigen selectively presented on the surface of cells, and after internalization, the cytotoxic payload is released when the linker is cleaved. This has been intensively explored to design novel therapies for a range of diseases^51^. ADCs have showed higher efficacy than unconjugated antibodies^52^ and have already been approved for the treatment of cancers such as relapsed Hodgkin and systemic anaplastic large cell lymphomas and many others are undergoing clinical trials^53^, thus making them a clinically relevant tool.

Here, we show that an ADC against a marker of the senescent surfaceome was able to selectively kill senescent cells with no toxicity to proliferating cells. This proves the feasibility of antibody-based targeted senolytics and suggests a new avenue for development tools that could be readily used for a wide range of therapeutic applications.

## Results

### B2M is a biological marker of senescence

**Figure 1:**
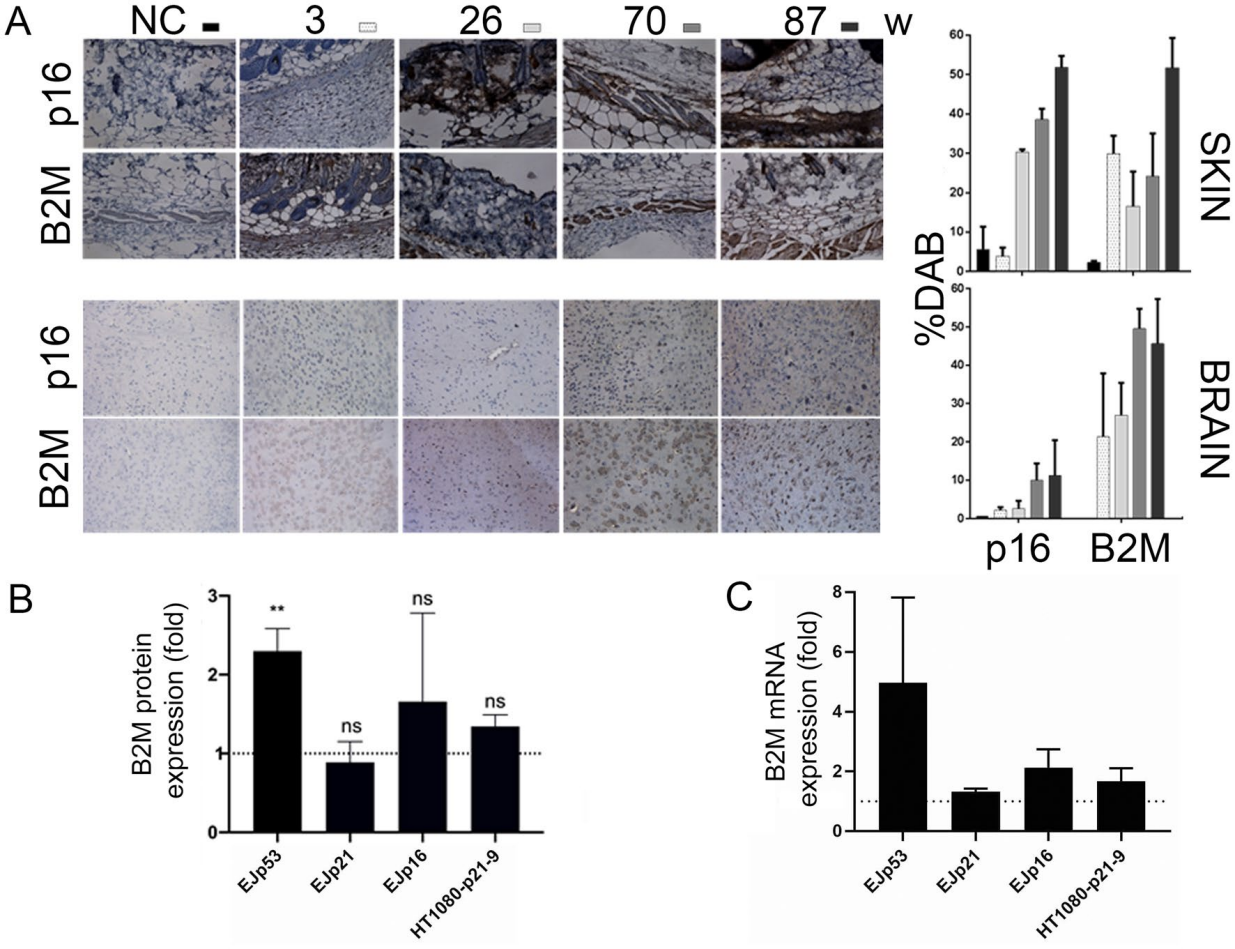
B2M expression increases with age in mice. (**A**) Representative images of **i**mmunohistochemical staining (left) of skin and brain (frontal lobe) samples from mice of different ages (3–87 weeks), showing B2M expression, as compared to p16 (senescence marker) Magnification: × 20. Graphs show quantitation of expression as %DAB (right). Bars represent the average of three independent samples for skin and five for brain, error bars represent standard deviation (SD). Fold change quantifications of B2M protein (**B**) and mRNA (**C**) expression levels, as measured by Western blot and quantitative real-time PCR, respectively, in EJp53, EJp21, EJp16 and HT1080-p21-9 cells induced to senesce for 4 days, normalized to the expression of their proliferating counterparts (represented by the dotted line at y = 1). Bars represent the mean ± SD of 3 independent experiments. ** *p* < 0.005 (compared to proliferating controls).

In order to develop new targeted senolytic strategies, we hypothesized that the proteins of senescent surfaceome that we recently identified^44^ could be used to specifically deliver toxic drugs into senescent cells^47^. To test this possibility, we selected a component of the MHC class I molecules, B2M, a membrane marker that we had previously validated^44,45^ and has a similar basal level of expression in most human tissues (Supplementary Fig. 1). We found that B2M protein levels increased progressively with age in brain and skin of mice, two tissues that present an obvious age-related functional degeneration, following a pattern similar to p16, a classic senescent marker^17,18^ (Fig. 1A). This suggests that B2M could be used in targeted senolysis to treat certain age-related pathologies, at least those manifested in these organs.

In order to explore this possibility, we used EJ bladder carcinoma cell lines with tetracycline (tet)-regulatable p53, p21 or p16 expression systems (EJp53, EJp21 and EJp16, respectively), which undergo senescence after tet removal^54–56^ (Supplementary Fig. 2). These are thoroughly characterized cellular models of senescence that uniquely allow for the interrogation of the main trigger pathways of senescence, without the interference of any other factor. As shown in Fig. 1B, there was a significant upregulation of B2M protein expression in EJp53. An RT-qPCR confirmed that the upregulation of B2M was also evident at the mRNA level in these cells (Fig. 1C). Of note, we did not observe any changes in B2M when senescence was induced by p21 overexpression in EJp21. This was confirmed using HT1080-p21-9, another well studied cellular model of p21-driven senescence^57^ (Supplementary Fig. 3), suggesting that B2M is induced in senescence through a p53-dependent mechanism that does not involve p21. We also observed a variable and non-significant p16-dependent upregulation of B2M in EJp16 at a protein level, which did not correlate with a induction at mRNA level . These data together confirm that B2M is induced in senescence in response to p53 , and perhaps moderately in response to p16 in certain situations. These are the two main pathways of induction of the phenotype, mainly representative of either stress-induced senescence (SIPS) and replicative senescence, respectively^58^, which suggests that B2M is a better marker for SIPS than replicative senescence.

### Development of a B2M-specific ADC

**Figure 2:**
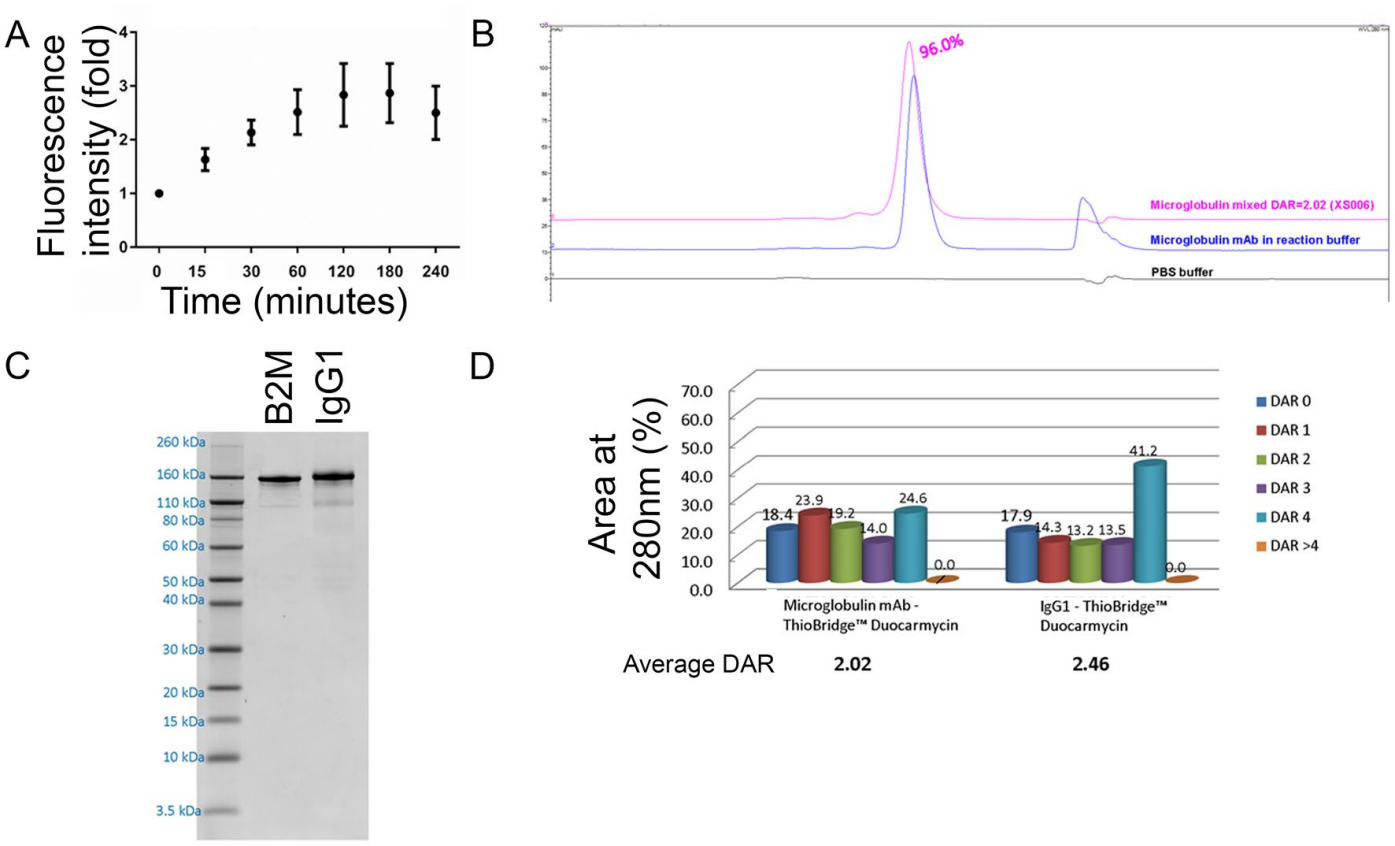
Characteristics of B2M-ADCs. (**A**) Internalization rate of the unconjugated B2M antibody in Control EJp16 cells as measured by CypHer5E fluorescence in a FACS analysis. Results show mean ± SD of three independent experiments. Results show mean ± SD of three independent experiments. (**B**) Size-Exclusion Chromatography characterization of the purity of the final ADC mix, compared to pure antibody and PBS. (**C**) SDS page comparing 1 µg of the unconjugated B2M antibody (UC) to an aliquot of the B2M ADC. (**D**) Quantitation of Drug-Antibody Ratios (DAR) of the B2M conjugated antibodies. Numbers indicate percentages of each DAR population in the mixture. Average DAR: 2.02.

To further explore whether targeted senolytics could be a clinically relevant possibility, we designed the first senolytic ADC. Of the five classes of human antibodies (G, A, D, E and M), the IgG isotype has been shown to have the lowest potential for immunogenicity^59^, and has thus been selected for most of the immunotherapeutics approved for clinical use (predominately the IgG1 subclass). As shown in Fig. 2A, we confirmed that a commercially available B2M IgG1 monoclonal antibody was internalized after binding to its target on the surface of senescent cells. To this end, we used a pH-sensitive cyanine dye method, CypHer 5, which is non-fluorescent at pH = 7.4 and maximally fluorescent at pH = 5.5, therefore being suited to report the movement of a receptor from the cell surface into internal acidic endosomes. Next, we conjugated this B2M antibody with duocarmycin, an irreversible DNA alkylating agent commonly used in ADCs^60–62^. The conjugation was established using an enzymatic cleavable linker. Through endo-lysiosomal trafficking, initiation of cleavage by the lysosomally active enzyme cathepsin B occurs when the antibody is internalized^63^ (Supplementary Fig. 4). The result was a B2M ADC with a 96% purity (Fig. 2B), a final concentration of 1.16 mg/ml (Fig. 2C) and an average drug/antibody rate (DAR) of 2.02 (Fig. 2D). An isotype IgG1 ADC also conjugated with duocarmycin was generated with similar characteristics (1.36 mg/ml, 2.46 average DAR and 93.2% purity) to be used as a control.

### Targeted senescent cell killing by an ADC against B2M

**Figure 3:**
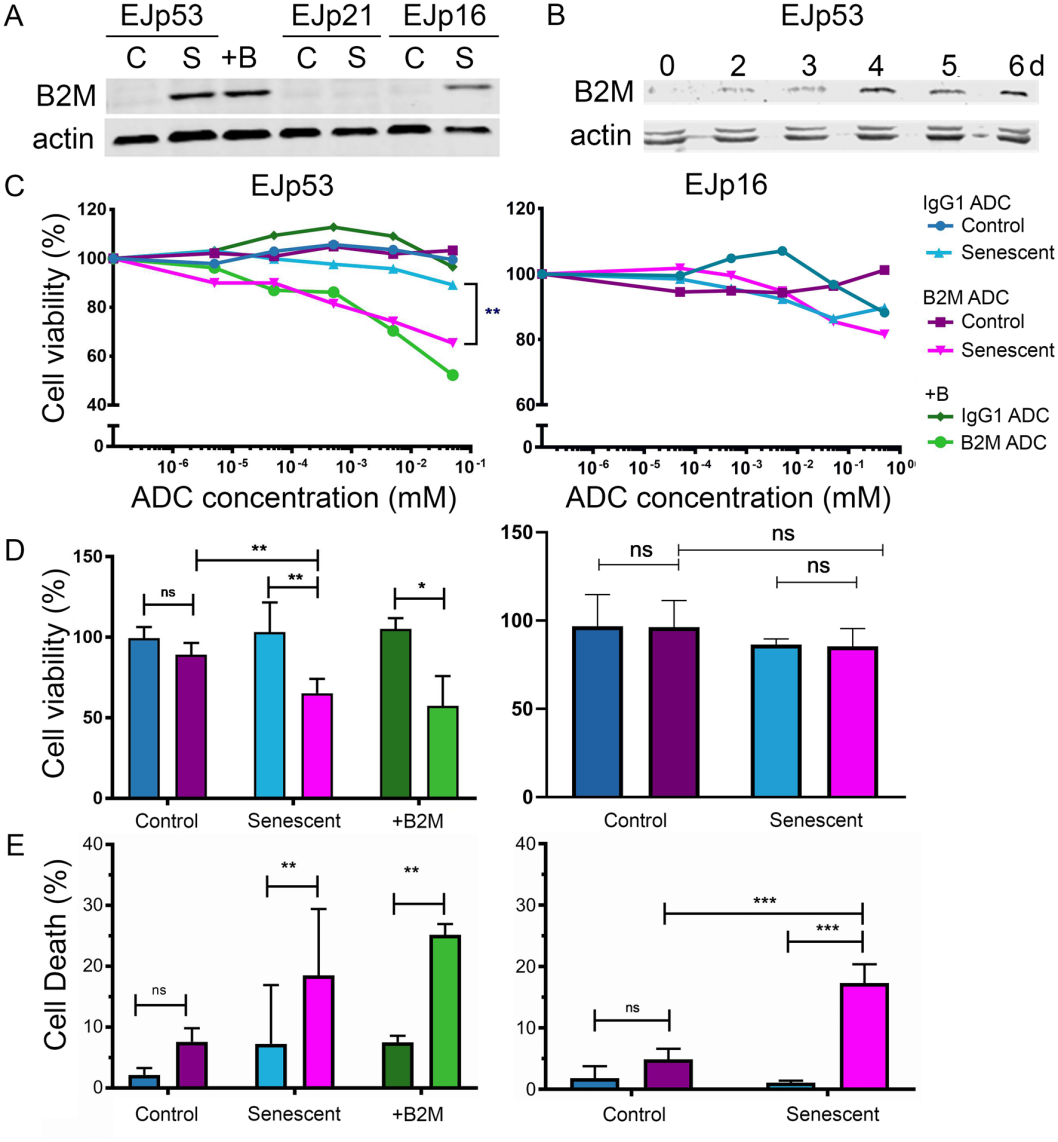
A B2M-duocarmycin ADC selectively kills senescent cells. (**A**) Representative Western Blot of B2M expression in EJp53, EJp21, EJp16 and HT1080-p21-9 cells 4 days after induction of p53 or p16 respectively, compared to EJp53 cells transfected with B2M cDNA for 4 days (+ B) . (**B**) Western blot showing B2M expression for different periods of time (0–6 days) in EJp53 cells transfected with B2M cDNA. (**C–D**) Cell viability, as measured by Cell Titre Glo, of proliferating and senescent (4 days after induction) EJp53 and EJp16 cells, incubated with different concentrations (C) or only 0.05 μM (bottom) of the B2M ADC or an isotype control for 72 h. An EJp53 cell transfected with B2M for 4 days was used as a positive control (+ B). Line and bar charts show the mean of 4 independent experiments. Colour codes match all panels. Bar represent the mean ± SD. * *p* < 0.05, ** *p* < 0.005. (**E**) Cell death, as measured by PI staining, in the same cells as above. Graphs show percentage of PI positive cells (dead cells). Bars represent the mean ± SD. * *p* < 0.05, ** *p* < 0.005.

To test the efficiency of the B2M ADC as a targeted senolyitic, senescence was induced in EJp53, EJp16 and EJp21 cells by removal of tetracycline. Also, non-senescent EJp53 cells were transfected with B2M to be used as a positive control. As shown in Fig. 3A,B2M was strongly expressed in response to p53 but not by p21, and only partially by p16, consistent with data shown in Fig. 1. Of note, p53-induced levels of expression of B2M were similar to those of the transfected positive control, which showed a consistent upregulation of B2M over six days, peaking at four days (Fig. 3B). We then incubated EJp53 and EJp16 with different concentrations of the B2M ADC or the IgG1 control ADC and measured cell viability by metabolic activity 72 h later. As shown in Figs. 3C,D, senescent EJp53 cells were sensitive to the B2M ADC but not to the isotype control, showing differences already at a nanomolar level, with the highest difference (65–89% viability) at 0.05 μM. Importantly, a similar difference was observed in the B2M-transfected positive control cells, confirming that the toxicity is dependent only on B2M expression. On the other hand, non-senescent proliferating EJp53 were not affected by the ADC, as expected from a senolytic agent. Of note, EJp16 were not sensitive to the B2M ADC, consistent with these cells expressing less B2M. These results were confirmed by measuring the amount of cell death by PI staining (Fig. 3E), which correlated with the cell viability data. As expected due to the lack of B2M expression, the B2M ADC had no cytotoxic effect in the p21-driven senescent models (Supplementary Fig. 5).

To confirm that the B2M ADC could successfully eliminate cells that undergo SIPS with high specificity, we treated HCT116 cells with the chemotherapeutic agent, doxorubicin. This is a classic model of a stress-induced p53-dependent senescent phenotype^64^ (see also Supplementary Fig. 6A). First, we observed that B2M was induced in HCT116 after senescence was established (Supplementary Fig. 6B), consistent with our hypothesis that B2M is a marker of SIPS. This suggested that these cells should be sensitive to the B2M ADC. Indeed, the ADC specifically induced a significant amount cell death in senescent HCT116 cells (Supplementary Fig. 6C), with a mean survival of 68% (compared to 94% in the non-senescent cells). This was similar to that observed in the EJp53 model. A specific Annexin V staining showed that the cell death induced by the B2M ADC was of not of apoptotic nature (Supplementary Fig. 6D), suggesting that necrosis could have been triggered instead. These results together show that a B2M ADC can be successfully used as antibody-based targeted senolytic, with significative cytotoxic effects seen in cells senescing as a result of activation of the p53 pathway (genetically or as a result of exposure to genotoxic drugs), and provides the first in vitro proof of the specificity and efficiency of this approach.

## Discussion

In the recent years, senescent cells have gained relevance as potential targets in the treatment for an ever-expanding range of diseases^6^. Indeed, the fact that the accumulation of these cells has been shown to play a significant role in a plethora of pathologies has sparked an interest in devising anti-senescence strategies, which could have a strong clinical impact, especially in age-related conditions^32^. To this end, there are three main approaches at reducing the negative impact of an excess of senescent cells in tissue homeostasis: inhibition of senescence before it occurs (senoblocking), clearance of already present senescent cells (senolysis) or inhibition of the negative functions of senescent cells (senostasis, also known as senomorphosis, mostly focused on inhibiting the various components of the SASP). The first two aim to solve the problem by preventing senescent cell accumulation, while the last one pursues the same final effect without affecting the ratio of senescent to normal cells.

The use of senoblockers as anti-ageing strategies has not yet been properly characterized. However, we have shown that interfering with the pathways that induce senescence can increase healthspan and lifespan in fast ageing mice without an increase in carcinogenesis^28^, which could be the most obvious unwanted effect of a senoblocker. This opens new avenues to be explored in the future. Senostatics are also in early phases of development, with no major milestones achieved yet. On the other hand, senolytics have been intensively researched and are already being studied in humans, with very positive preliminary results. Because of this, the race to develop senolytics is advancing at a fast pace, simultaneously following different avenues. While inhibitors of the BCL2 proteins and other small chemicals have so far proven to be the most effective in preclinical studies^21,33^ and cell-based strategies have also been proposed^65^, there is still a need for more targeted approaches with higher specificity and efficacy that the current ones.

A key limiting factor for this is the ability to identify senescent cells, due to the lack of markers that are specific and universal. To address this issue, we screened for membrane proteins upregulated during senescence that have extracellular epitopes, and validated a series of novel markers in what was the first characterization of the senescent surfaceome^44^. Since then, the surfaceome has emerged as a potential source of targets for senolysis and senostasis^66^. Here, we tested ADCs as a strategy that takes advantage of the senescent surfaceome to specifically deliver toxic payloads into senescent cells. Although this is a novel approach to clearing senescent cells, a similar idea has already been developed and validated in the treatment of cancer^53^, which suggests that this form of targeted senolysis could potentially be useful and could shorten the time needed to reach clinical trials.

ADCs are based on the recognition by an antibody of an extracellular epitope, after which the antibody is internalized and the drug attached to it is released by cleaving of the linker molecule in the lysosome^67^. They have been successfully developed to target a variety of cancer cells^52^. The first ADC approved for clinical use was Gemtuzumab ozogamicin, in 2000, followed by brentuximab vedotin and ado-trastuzumab emtansine^53^. Several ADCs are currently used in the clinic (a total of nine, to the best of our knowledge: Mylotarg, Adcetris, Kadcyla, Besponsa, Polivy, Enhertu, Padcev, Trodelvy and Blenrep), and there are many others under development, using targets such as CD22, CD33 or CD138^68^.

Consistent with our previous results using B2M-targeted nanoparticles to kill senescent cells^47^, our experiments show here that a B2M monoclonal antibody linked to duocarmycin selectively induces the death of senescent cells, without importantly affecting the survival of the control proliferating cells. Moreover, B2M-negative senescent cells did not respond to the ADC, and an isotype control antibody did not have an effect on senescent cell survival, which shows that the drug delivery occurs indeed through specific binding of the antibody to B2M and does not affect cells with low expression of the marker. Importantly, we saw a toxic effect in cells induced to senesce by either p53 or p16, the two main pathways involved in triggering the phenotype, suggesting that the ADC could have an effect on a wide range of senescent cells. However, the induction of cell death was evident only in p53-induced senescence, suggesting that B2M could be a more relevant target for SIPS, which relies more on this pathway. This is confirmed by the data showing cell death in the HCT116 model of chemotherapy-induced senescence, a well-studied example of SIPS. Conversely, the lack of response in p16-driven senescent cells suggests that the B2M antibody would be less effective in clearing replicative senescent cells, which is a phenotype in which activation of the p16 pathway features prominently. Indeed, preliminary experiments indicated no effect on replicative senescent fibroblasts (data not shown). These results highlight the relevance of the models we used, which, although artificial, are the only approach that allows for independent interrogation of the distinct pathways of senescence and thus provide a general knowledge that can then be applied to other forms of senescence, both in vitro and in vivo. Also, this information suggests that ADCs could be made against differ targets for more selective killing of replicative or stress-induced senescence, depending on the clinical needs, and even make them cell type- or tissue-specific. A comparison of the effects to those of a panel of known senolytics in different models of senescence could also be useful to anticipate the in vivo models that could benefit from this ADC.

These proof-of-principle data show that ADCs could be used effectively to clear senescent cells. More experiments will need to be performed to fully understand the mechanisms involved. Although our data strongly suggests that cell death is achieved by internalization of the ADC and the subsequent cleavage of the linker that releases the toxic payload and induces cell death (Supplementary Fig. 4), alternative pathways that may also contribute to the effect would have to be explored. It is important to note that the choice of senescent marker for such ADCs is likely to have an effect on the specificity of the treatment. Background expression in tissues will have to be carefully considered. Because of this, B2M may not be the target of choice, due to a widespread basal level of expression (see Supplementary Fig. 1), although specific delivery to a tissue of choice, such as brain or skin (see Fig. 1), could greatly reduce any systemic effects. In vivo experiments will be needed to determine if this is an obstacle to use such an ADC in humans or there is a wide enough therapeutic window. Also, the B2M ADC could be particularly useful to clear the senescent cells that arise after chemo/radiotherapy, a form of SIPS, which are suspected to be involved in tumour resistance and relapse^69^. Others could be tailored to address issues related to accumulation of cells due to replicative senescence, which play an important role in age-related pathologies, by using a different epitope. If necessary, cytotoxic stimuli that require the presence of more than one target on the cell surface could be designed (for instance, a toxic payload divided in two essential parts^19^, which could be delivered into cells using ADCs against two different markers). This would greatly reduce the potential toxic side effects while increasing their specificity and thus increasing the feasibility of the approach.

Preliminary experiments in animal models suggest that clearing senescent cells should have a positive effect on healthspan and lifespan^19,20,37^, fibrosis^70^, hepatic steatosis^23^ , vasomotor dysfunction^71^, pulmonary fibrosis^72^, osteoarthritis^72^ and could improve the response to cancer therapies^73^. Our results indicate that antibodies could be an efficient system to bring toxic drugs into specific types of senescent cells in humans with minimal side effects, following up on the success of similar approaches in cancer treatment. Further studies will be needed to determine the best targets, as well as the safety and effectivity of the different delivery options.

## Materials and methods

### Cell culture, treatment and senescence induction

All cells were cultured in Dulbecco’s Modified Eagle’s Medium (DMEM) supplemented with 10% Foetal Bovine Serum (FBS), 100 IU/ml penicillin and 100 μg/ml streptomycin. Cells were incubated at 37 °C in a humidified incubator with 5% CO_2_. To keep all the different EJ cells proliferating, 1 µM tetracycline was added to the culture media and this was replaced every three days. EJp16 cells were maintained in complete culture media supplemented with 100 μg/ml hygromycin and 2 μg/ml puromycin. EJp53 and EJp21 cells were maintained in culture media supplemented with 100 μg/ml hygromycin and 750 μg/ml geneticin. To induce senescence by tetracycline removal, cells were trypsinized, washed with 1 × phosphate buffered saline (PBS) and centrifuged at 1100 rpm for three minutes. This wash step was carried out three times. All three EJ cell lines were generated in the lab of Stuart A. Aaronson and were provided as a gift. To induce p21 expression in HT1080-p21-9, 100 μM of isopropyl β-D-1-thiogalactopyranoside (IPTG) was added to the medium. HT1080-p21-9 were a generous gift of I. Roninson. To induce senescence in HCT116, cells were exposed to 1 mg/ml doxorubicin for 4 days. HCT116 were a generous gift of B. Vogelstein. Once senescent, cells were exposed to different concentrations of the control and B2M ADCs for 72 h, before cell viability or cell death were measured. A Bio-Rad TC20 Automated Cell Counter was used in all cell counting experiments and before seeding the cells.

### Western blot analysis

For lysate extraction, the medium was removed from cells and plates were washed once with 1 × PBS. Cells were trypsinized, collected, centrifuged and the pellets kept on ice. The cell pellet was re-suspended in 100 µl of ice cold RIPA lysis buffer (radio immunoprecipitation assay—150 mM NaCl, 50 mM Tris HCl pH 8.0, 1% NP40, 0.1% SDS, 0.5% sodium deoxycholate) containing a 1 μg/ml Protease and Phosphatase Inhibitor Cocktail Set III (Calbiochem) and incubated for 20 min on ice. Cells were ruptured by passing through a syringe 10 times or with sonication, and centrifuged at 14,000 g for 15 min at 4ºC. The supernatant was transferred into Eppendorf tubes and protein concentrations were determined using the Bradford protein assay (Fermentas). 4X Laemmli buffer was added in 1:4 ratio and samples were heated at 95 ºC for 5 min. 20 μg of total protein per sample were subjected to 10% or 15% SDS-PAGE and transferred to Immobilon-P membranes (Millipore). An Odyssey CLx Infrared Imaging System (Li-COR, Lincoln, NE, USA) was used to visualize the results. Primary antibodies used were: β-actin (Abcam, #ab8227), p16 (Abcam, #ab54210), p21 (Santa Cruz Biotechnology, #sc-53870), p53 (Santa Cruz Biotechnology, #sc-126 ) and B2M (LifeSpan BioSciences, #LS-B2200). Original, un-processed images of the Western blots are presented in Supplementary Fig. 7. For the blot in Figs. 3A, S2 and S3, the original image could not be located due to a technical failure in the storage media and a replicate blot has been shown instead.

### Senescence-associated β-galactosidase staining

Senescence associated β-Galactosidase (SA β-Gal) staining was performed as previously described^74^. Briefly, cells were fixed with 10% neutral buffered formalin and incubated with staining Solution (1 mg/ml X-gal in dimethylformamide, 40 mM Citric Acid/Na Phosphate Buffer, 5 mM Potassium Ferrocyanide, 5 mM Potassium Ferricyanide, 150 mM Sodium Chloride, 2 mM Magnesium Chloride in distilled water). Plates were then incubated at 37ºC in a non-CO_2_ incubator and observed after 12–16 h.

### B2M expression

In order to express B2M, a B2M cDNA (OriGene) was transfected into EJ cells using Lipofectamine 2000 reagent (Invitrogen), following manufacturer’s instructions, in a 1 µg: 2.5 µl ratio. Cells were incubated at 37ºC for 6 h after which the growth media was changed to complete DMEM.

### Antibody internalization

To measure the internalization of the B2M antibody (LifeSpan BioSciences, #LS-B2200) after binding to its target, we used a CypHer5E kit (GE Healthcare), following manufacturer’s instructions. Briefly, cells were collected gently by scraping before washing with cold PBS and kept on ice. Next, cells were centrifuged at 200xg for 5 min at 4ºC. The pellets were resuspended in Blocking Buffer (0.5% BSA in 1xPBS) and incubated for 15 min on ice and spun down 500xg for 5 min in 4ºC. The supernatant was discarded, and the cells were incubated in time-dependent manner with primary antibodies conjugated with CypHer5E Mono NHS Ester. The conjugation of CyperHer5E Mono NHS Ester to an antibody was performed following manufacturer recommendations. Then, the cells were washed 2 times with Blocking Buffer and spun down. The pellets were resuspended in the Blocking Buffer and the internalisation was read using FACS on the APC channel.

### Synthesis of ThioBridge reagent

To a stirred solution at 0 °C of 4-((S)-2-((S)-2-((tert-butoxycarbonyl)amino)-3-methylbutanamido)-5-ureidopentanamido)benzyl-(1-(chloromethyl)-3-((E)-3-(4-methoxyphenyl)acryloyl)-2,3-dihydro-1H-benzo[e]indol-5-yl)carbamate (Boc-val-cit-PAB-duocarmycin; 36 mg) in DCM (3 mL) was added TFA (1.5 mL). After 1 h, the DCM was removed in vacuo and the residue dissolved in a solution of (1-[Bis(dimethylamino)methylene]-1H-1,2,3-triazolo[4,5-b]pyridinium 3-oxid hexafluorophosphate) (HATU) (4.1 mg) in DMF (1 mL), under an argon atmosphere and cooled to 0 °C. To this was added the known bis-tolylsulfonyl-propanoyl-benzamide-L-Glu-[OH]-[PEG(24u)-OMe]^75^ (70.0 mg) and HATU (22.3 mg) in dry DMF (1 mL) at 0 °C followed by the addition of N-methylmorpholine (NMM) (13.2 µL). After 1.5 h, the reaction solution was concentrated in vacuo, dissolved in acetonitrile (1 mL) and purified by reverse phase C18-column chromatography eluting with buffer A (v/v): water:5% acetonitrile:0.05% trifluoroacetic acid and buffer B (v/v): acetonitrile:0.05% trifluoroacetic acid (100:0 v/v to 0:100 v/v). The organic solvent was removed in vacuo and the aqueous solvent was removed by lyophilisation to give the bis-tolylsulfonyl-propanoyl-benzamide-Glu-[NH-PEG(24u)-OMe]-[val-cit-PAB-duocarmycin] ThioBridge reagent as a thick clear colourless oil (45 mg).

### Synthesis of ThioBridge ADC

Conjugation of reagents to a monoclonal (mAb) IgG1 B2M Antibody (LifeSpan BioSciences, #LS-B2200) and IgG1 Isotype (non-binding) mAb was performed by adding tris(2-carboxyethyl)phosphine (TCEP) (1.5 equivalents per inter-chain disulfide) to a solution of the antibodies (5.2 mg/mL, 20 mM sodium phosphate, pH 7.5, 150 mM NaCl, 20 mM EDTA). Following incubation at 40 °C for 1 h, the reduced antibodies were cooled to 22 °C and diluted to 4.4 mg/mL using additional phosphate buffer. The ThioBridge reagent was dissolved in a mixture of DMF and propylene glycol (1:6). 1.5 equivalents of ThioBridge reagent per inter-chain disulfide was added to the reduced antibody solution to give a final mixture containing 5% DMF and 30% propylene glycol /v/v which was then incubated at 22 °C for 18 h. The crude conjugates were purified by preparative size exclusion chromatography using a HiLoad 16/60, Superdex 200 prep grade column giving an average DAR loading of 2 for the ThioBridge 2M2 ADC and 2.5 for the ThioBridge IgG1 non-binding ADC.

### Quantitative real-time PCR

Total RNA was extracted from 10^6^ cells using the ReliaPrep Cell Total RNA Miniprep System Kit (Promega), following manufacturer’s recommendations. The concentration and purity of extracted RNA was measured using a NanoPhotometer P300 (Implen) and cDNA synthesis was performed using the Superscript III First-Strand Synthesis System for RT-PCR (Invitrogen), according to manufacturer instructions. Quantitative real-time PCR was performed using SensiMix SYBR-No-Rox (Bioline) in a LightCycler 480 (Roche) as previously described^76^. For the primers used, see Supplementary Table 1. Results were analysed on Microsoft Excel using the comparative ΔΔCt method and graphs were plotted using GraphPad Prism 7.0 Software.

### Cell viability and cell death measurements

Cells were seeded into 96-well plate with a final density of 4 × 10^5^ cells/ml. To assess cell viability after the treatment, CellTiter-Glow (Promega) reagent was added into each well and luminescence was recorded using a Hidex Sense multimode microplate reader. Cell viability was calculated using Microsoft Excel and plotted using GraphPad Prism 7.0 Software. The percentage of cell death was assessed using Propidium Iodide (PI) staining. First, cells were fixed with 70% ethanol and incubated for 30 min in the dark in PI staining solution (50 µg/ml Propidium iodide, 10 µg/ml RNase A in 1 × PBS). Alternatively, apoptosis was measured by Annexin V/PI staining, as previously described^77^. Flow cytometry analysis was performed using the BD FACSCanto II (Becton Dickenson Biosciences) and the results were analysed using FACS Diva 6.1.3 software (BD Bioscience) and the GraphPad Prism 7.0 software.

## Supplementary Information

Supplementary Information.
