# Supplementary material for: Targeted clearance of senescent cells using an antibody-drug conjugate against a specific membrane marker
Source: Sci Rep. 2021 Oct 13;11:20358. doi: 10.1038/s41598-021-99852-2 (PMC8514501; doi:10.1038/s41598-021-99852-2)
Supplement: Supplementary file 1 — Supplementary Information. [file 41598_2021_99852_MOESM1_ESM.docx]

**Targeted clearance of senescent cells using an antibody-drug conjugate against a specific membrane marker**

Marta Poblocka, Akang Leonard Bassey, Victoria M. Smith, Marta Falcicchio, Ana Sousa Manso, Mohammad Althubiti, XiaoBo Sheng, Andrew Kyle, Ruth Barber, Mark Frigerio and Salvador Macip

**SUPPLEMENTARY INFORMATION**


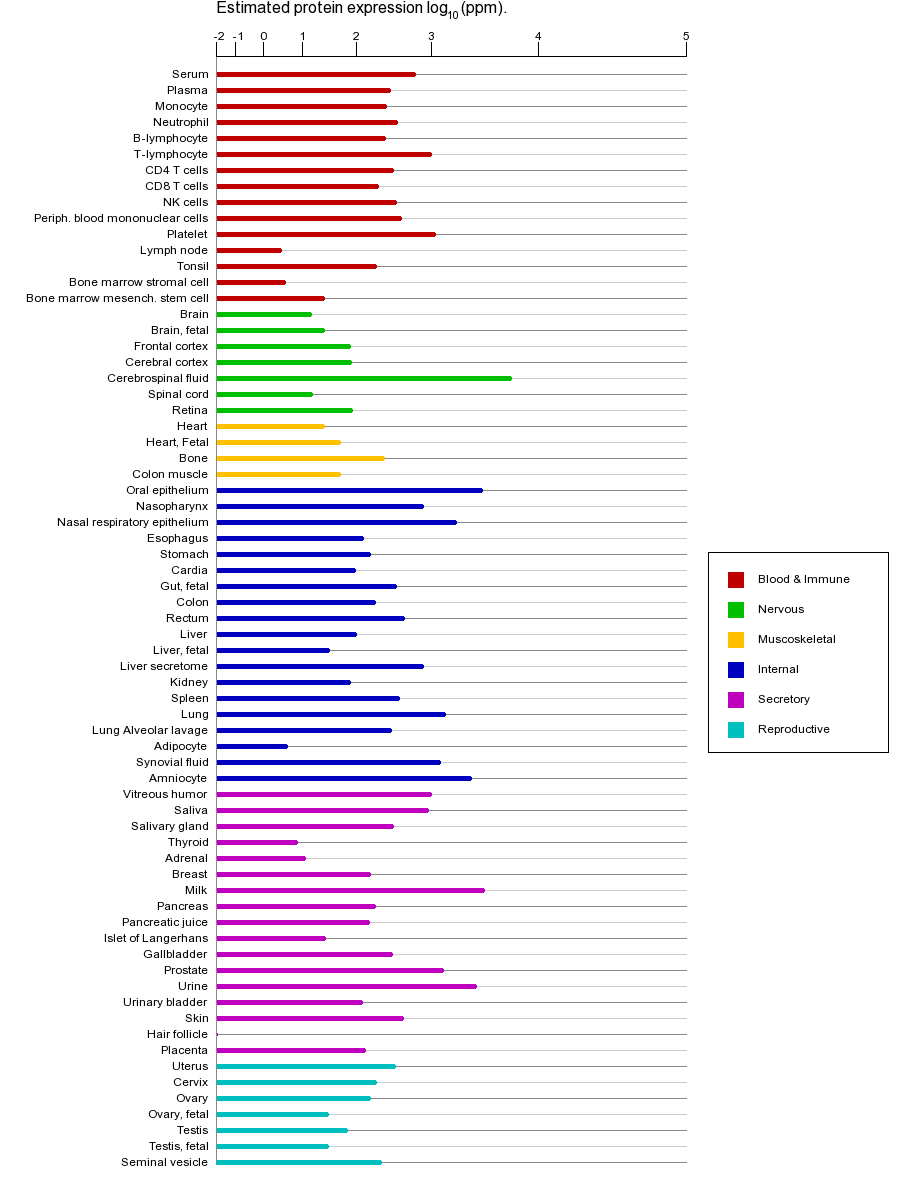


**Supplementary Figure 1.** Estimated basal B2M protein expression in different human tissues. Data obtained from GeneCards.org, from ProteomicsDB, PaxDb, and MOPED.

**
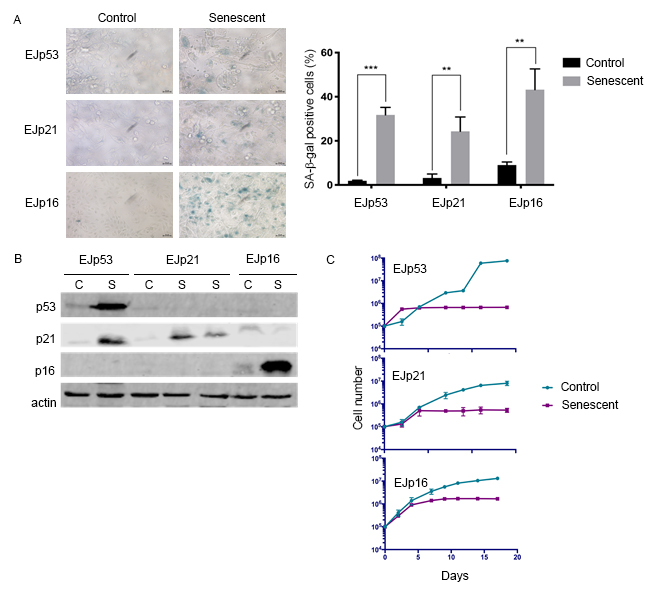
**

**Supplementary Figure 2.** **Induction of cellular senescence in EJp53, EJp21 and EJp16** **cells**. **(A)** Representative images of SA-β-Gal staining (left) and its quantification (right) of EJp53, EJp21 and EJp16, uninduced (Control) or 4 days after tet removal to induce exogenous p53, p21 and p16 expression, respectively (Senescent). Blue colour indicates positive staining. Bars represents mean values ± SD of 3 independent experiments. ** p<0.005; *** p<0.0005. **(B)** Representative Western blot of p53, p21 and p16 expression in the same cells to confirm the specific induction of these proteins, 4 days after tet removal (C: control; S: senescent). B-actin was used as a loading control. **(C)** Proliferation of EJp53, EJp21 and EJp16 as measured by cell counting after induction of exogenous p53, p21 and p16 by tet removal, confirming that senescent cells do not proliferate. Graphs represent mean values ± SD of 2 independent experiments.


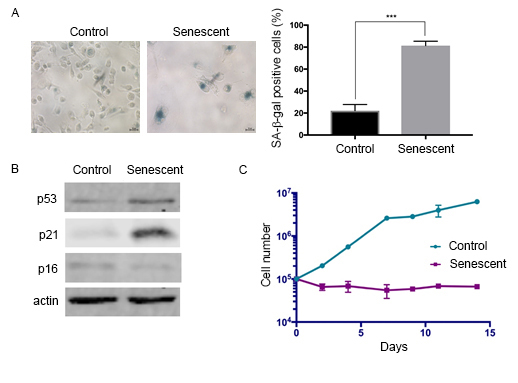


**Supplementary Figure 3.** **Induction of cellular senescence in HT1080-p21-9.** **(A)** Representative images of SA-β-Gal staining (left) and its quantification (right) of HT1080-p21-9 uninduced (Control) or 4 days after addition of IPTG to induce exogenous p21 expression (Senescence). Blue colour indicates positive staining. Bars represents mean values ± SD of 3 independent experiments. *** p<0.0005. **(B)** Representative Western blot analysis of p53, p21 and p16 expression in HT1080-p21-9 without induction (Control) or 4 days after induced expression of exogenous p21 (Senescence). B-actin was used as a loading control. **(C)** Proliferation of HT1080-p21-9 cells, as measured by cell counting after induction of exogenous p21 by addition of IPTG, confirming that senescent cells do not proliferate. Graphs represent mean values ± SD of 2 independent experiments.

**
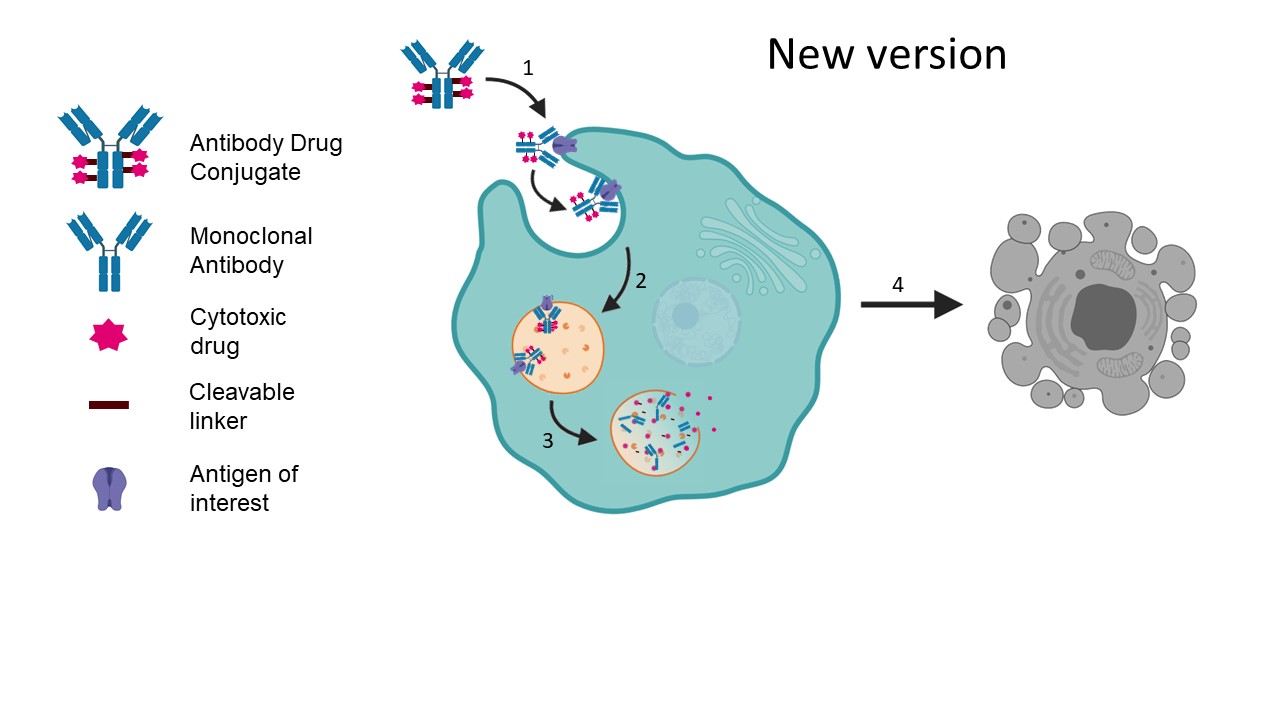
**

**Supplementary Figure 4.** **Structure of ADCs and their proposed mechanism of action.** Antibody drug conjugates (ADCs) are comprised of a monoclonal antibody conjugated to a cytotoxic compound through chemical cleavable linker. ADCs recognize and bind to a specific antigen of the surfaceome (1). The ADC-antigen complex is internalized and trafficked into lysosomes (2). In there, ADCs are lysed and the cytotoxic payload is released (3), leading to cell death (4).


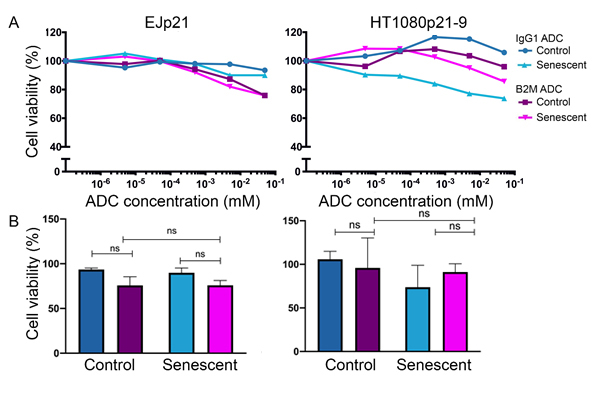
 **Supplementary Figure 5.** **The B2M ADC has no effect on control and senescent cells lacking B2M protein expression**. Cell viability, as measured by Cell Titre Glo, of proliferating and senescent EJp21 and HT1080p21-9 cells 5 days after induction of p21 expression, incubated with different concentrations **(A)** or only 0.05μM **(B)** of the B2M or IgG1 ADCs for 72 hours. Colour codes match all panels. Line charts show the mean of 3 independent experiments. Bar charts represent the mean ± SD of 3 independent experiments. No differences were statistically significative.


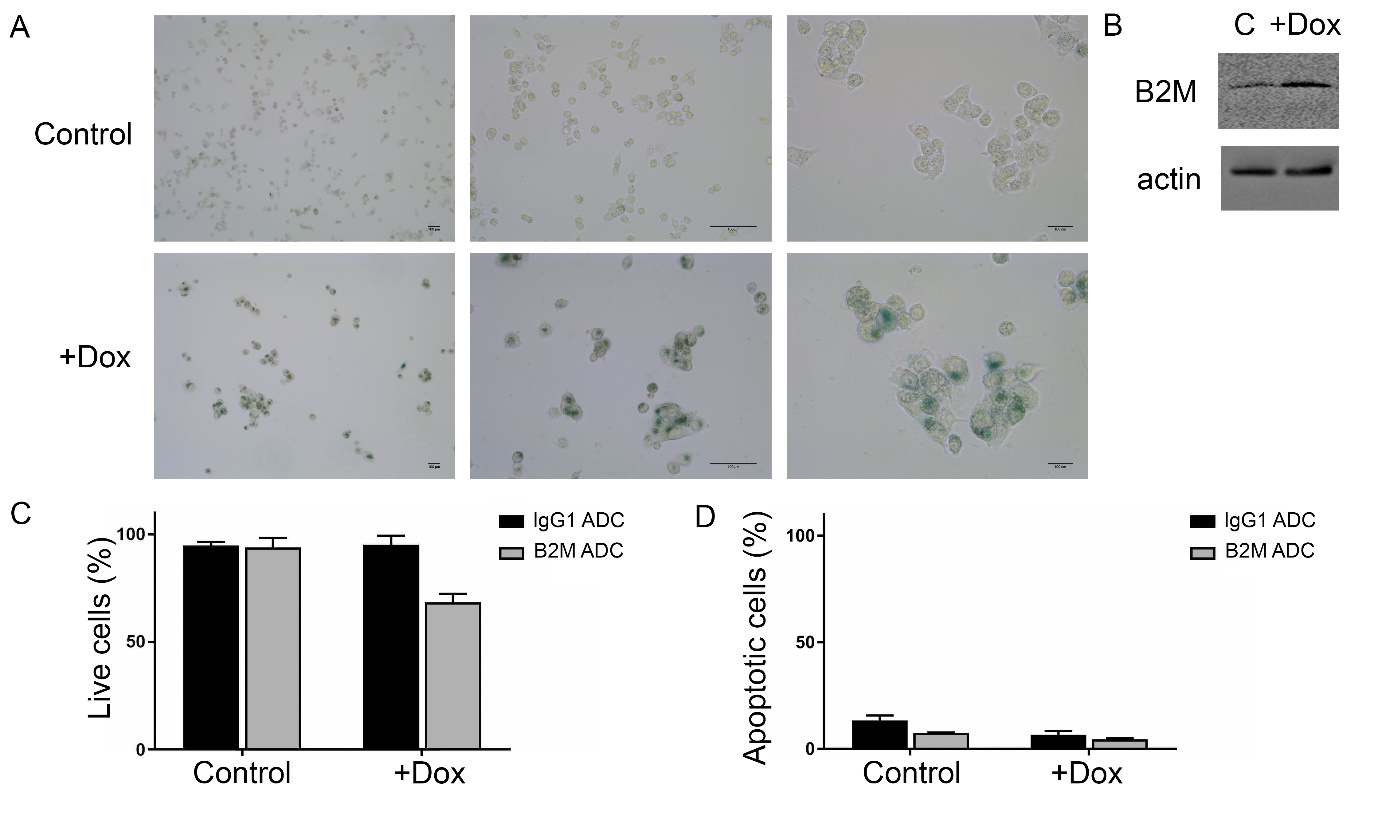


**Supplementary Figure 6.** **The B2M ADC specifically induces cell death in HCT116, a model of SIPS.** HCT116 cells were treated with 1mg/ml doxorubicin to induce senescence**. (A)** Representative images of SA-β-Gal staining of HCT116 exposed (+Dox) or not (Control) to doxorubicin for 4 days. Each column represents a different magnification. **(B)** Representative Western blot analysis of B2M expression in the same cells. **(C)** Percentage of live cells in HCT116 control and treated with doxorubicin, and then exposed to 0.05μM of the isotype ADC or the B2M ADC, as measured by PI staining. Plots represent mean and standard deviation (n=2) of PI negative cells normalized to the controls (cells not treated with any ADC). **(D)** Percentage of Annexin V positive/PI negative cells (apoptotic) in the same samples. Plots represent mean and standard deviation (n=2). No differences were statistically significative.

**
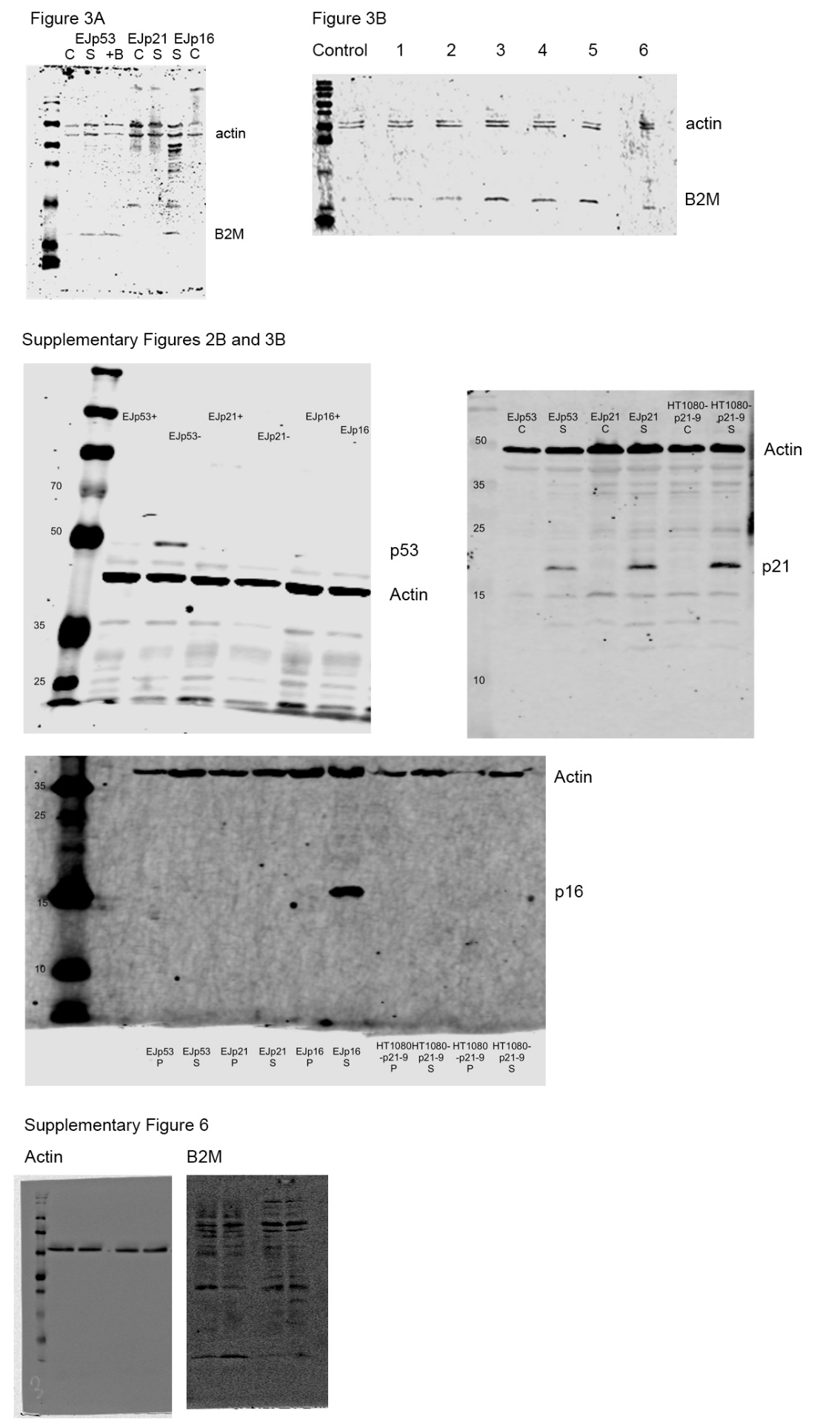
**

**Supplementary Figure 7.** Full representative Western blots of the experiments in the previous figures.

| Gene | Primer sequence (5’- 3’) | Harvard Primer bank ID: |
| --- | --- | --- |
| p16 | F: 5’ GATCCAGGTGGGTAGAAGGTC 3’  R: 5’ CCCCTGCAAACTTCGTCCT 3’ | 17738298a1 |
| p53 | F: 5’ CAGCACATGACGGAGGTTGT 3’  R: 5’ TCATCCAAATACTCCACACGC 3’ | 371502118c1 |
| p21 | F: 5’ TGTCCGTCAGAACCCATGC 3’  R: 5’AAAGTCGAAGTTCCATCGCTC 3’ | 310832423c1 |
| B2M | F: 5’ GAGGCTATCCAGCGTACTCCA 3’  R: 5’ CGGCAGGCATACTCATCTTTT 3’ | 37704380c1 |
| GAPDH | F: 5’ TCTCTGCTCCTCCTGTTC 3’  R: 5’ GCCCAATACGACCAAATCC 3’ | N/A |

**Supplementary Table 1.** Primers used for RT-qPCR.
